# Medicine and Surgery in the Twentieth Century*Kansas State Homœopathic Society, 1896.

**Published:** 1896-07

**Authors:** Henry W. Roby

**Affiliations:** Topeka, Kansas


### MEDICINE AND SURGERY IN THE TWENTIETH CENTURY.*

HENRY W. ROBY, M. D., TOPEKA, KANSAS.

" Watchman, tell us of the night,
What it's signs of promise are.
Watchman, tell us of the dawn,
What shall be its guiding star."

In the unfolding of the fleeting centuries that are rushing from a great unknown inception to a great unknown conclusion, each century and many of its subdivisions present to the human mind majestic and astonishing problems for solution. In the coming century, whose glow of dawn we already see, lie coiled and struggling to be unfolded the mightiest problems that ever appealed to mortality. The struggle is upon us already to make that clause in the Declaration of Independence, that "all men are created free and equal," the reigning reality among all men. More human hands than man can count are clutching

* Kansas State Homœopathic Society, 1896.

and tugging at the car of progress, to drag it in triumph over the stony paths of life, just as a few years ago the votaries of Vishnu clutched the great car of Juggernaut, to roll it in superstitious triumph over the trembling earth and the prostrate forms of wild devotees.

All along the highway of human advancement willing hearts and willing hands are toiling through sunlit days and sleepless nights in the dream and hope of bettering the condition of mankind. The law-giver is alert to formulate the best laws on all the statute books. The philanthropist is calling on all men to aid him in uplifting the poor in heart as well as in purse. The inventor is racking his brain and wearing out soul and body in a grand struggle to invent machines and devices to lighten human labor and multiply human blessings. The press and the pulpit are thundering like a tornado over the great, flinty road to universal betterment, and in their wake the shrines and temples of wrong and oppression are toppling and crashing to everlasting ruin. That subtle servitor, electricity, is already bearing the burdens of countless men and women up the rugged slopes of life, while they ride toward the summit in its swift car and in the glare of its dazzling light. The reapers of the world's harvest ride leisurely upon clanking machines that do the work of untold laborers, and there is seen all over the face of the earth a vim and push along the lines of progress that, were it reversed and directed against humanity, would be appalling and paralyzing. Patrick Henry truly said there is no way of judging the future except by the past, and applying that rule of judgment, what may we not expect along all the lines of human endeavor in the next century? If every other human interest is advancing, not at a snail's pace, but upon the swift wings of the lightning, may we not surely calculate on great progress in medicine and surgery? Think what has been done along the lines of chemistry, pharmacy, surgery, obstetrics, bacteriology, and therapeutics in the very few centuries that have already pitched their tents on the eternal camping-ground of the ages! In the single century that is now closing what wonderful strides has the *genus medicus* taken.

"Science for centuries devoted itself to the cataloguing of facts and the discovery of laws. Each worker toiled in his own little place—the geologist in his quarry, the botanist in his garden, the biologist in his laboratory, the astronomer in his observatory, the historian in his library, the archæologist in his museum. Suddenly these workers looked up; they spoke to one another; they had each discovered a law; they whispered its name. It was Evolution. Henceforth their work was one, science was one, the world was one, and mind, which discovered the oneness, was one," and that *one* entity, mind, was the progressing, moving entity of the world which has dominated all the past centuries as well as the present, and will dominate the centuries to come.

Two hundred years ago medicine presented a greater babbling and confusion of tongues than the great tower of Babel. Within that time, revolution after revolution in therapeutic methods has occurred, and each revolution was but a phase of evolution. Thought quickened, perception grew broader and deeper, conception of powers and capacities of principles and relationships grew greater, profounder, until out of the chaos came order, harmony, system, consistency, and a more universal success. Great discoveries were made, great skill attained, and great results achieved.

Now, march all these analytical and constructive forces forward into the new century and add to them the genius and the multiplied advantages naturally coming over into this field from all the other fields of science, and see what an avalanche of forces shall march in triumph through the twentieth century. With the mighty forces of chemistry, electricity, magnetism, the microscope, the spectroscope, the telescope, the culture tube, and the weather bureau all harnessed to the car of medicine and guided by an ever-increasing intelligence and an ever-growing warmth of human sympathy and loving solicitude, and who can adequately predict the triumphs and victories in this great field of contest? We may justly hope and predict that the stubborn and arrogant conflict of dogmatism will have passed away, and that all medical men will dwell together in unity. That no man will be ostracized by his fellow-laborers in humanity's cause for believing that drugs have one or two modes of action. That increased light and knowledge will enable medical men to serve the world more acceptably in the role of sanitarians than of phlebotomists; that the chief function of the physician will be to teach people how to live wholesome and untainted lives, and to die of old age instead of pestilence. That when drugs become necessary at all against accidental disorders, men will be wise enough and sufficiently familiar with the action and powers and properties of drugs to make them serve their benign purpose in such minute quantities that no life shall be put in peril of the dose. That all the appliances for the mitigation of bodily ills shall be as delicate as are now the most sensitive chronometers and microscopes. That hygienic and vital knowledge will be so general and diffuse that it will first be a disgrace, then a misdemeanor, and then a crime to be ill of a preventable disease, and the medical man's status in the community, his reputation and honor will depend upon his ability to teach his patients, both by precept and example, the pure gospel of health, which, like a great benediction, shall cover every clime and race and tongue on the planet. Such we may confidently expect to be the state of medicine in the twentieth century.

Now, walk with me down the path of all the sciences and we shall see that none of them are brighter with hope and blessings for the race than the surgeon's path. It will still pass by some new-made graves, but they will grow fewer the further along we walk in it. To-day humanity has no better friend, and nothing on earth so near to a savior as the well-equipped surgeon. When your untutored hand and brain fail to rescue your wife or child from impending death; when your kind and humane neighbors all fail after using their best efforts; when your family physician, with all the aids that materia medica and sanitary science affords, fails; when your minister and Church and Christian science and faith cures all fail, and death seems to have won the fight and is just in the act of carrying off your loved one, the true, skillful, and brave surgeon often steps in and says to death, "Hold, stay thy hand!" and to the broken-hearted says, "Have courage, there still is hope," and to the bystanders says, "Be brave and lend a hand," then, with the courage of a lion and nerves of steel, and a heart as tender as a woman's, he lays the glittering blade close alongside the false growth that is sapping the life of your loved one and draws its keen edge close between quivering nerves and throbbing arteries close down among the well-springs of life, and with a master hand severs the false from the true flesh and casts the monstrous growth loose from the body it had so nearly slain, or lets the sweet air of heaven into croup-shut lungs, and then gently and swiftly takes up the severed and bleeding vessels and secures them against further loss of the life current, dresses the new-made wound, and then sits by the side of your loved one with finger on pulse, and eyes and ears and all his powers alert for any lurking or approaching danger, and stands by, without food, without sleep, without rest, in as deep concern for the life that is in peril as you feel, and then when death, baffled and beaten, skulks away, and he restores to you alive and well and free from peril your loved one, he becomes the *summum genus* of the race.

It is a marvelous fact that many thousands of women are alive and well to-day from whose bodies the surgeon's knife has severed tumors weighing from one to one hundred pounds, ninety-seven out of a hundred of them saved from otherwise inevitable death.

Even in this twilight hour of the nineteenth century, we almost ruthlessly invade the sacred precincts of the pelvis, abdomen, chest, and skull and cast out the offending neoplasms, exsect retrograding tissues, and resect and anastomose tubular structures with surprising and gratifying results. That which was once a forbidden shrine is to-day the daily camping-ground of the surgeon, and enterorraphy, ovariotomy, oöphorectomy, and hysterectomy are rapidly shifting from the field of formidable and perilous procedures, and being relegated to the realm of minor operations.

With all the analytical and corrective operations that the mind can formulate already accomplished facts, what have we left for the next century to solve but the synthetical and constructive procedures? Will we not then graft legs and arms and hands and feet as easily as we now graft skin? Will we not then transplant sections of lungs and hearts and brains as now we do sections of bones decalcified? Shall we not then pluck the good eyes from the young victim of a fatal traumatism and hastily transplant them to the sockets of the octogenarian as readily and successfully as we now transplant apple trees from the nursery to the garden? Shall we not yet establish a new optic commissure, when the old one is destroyed, with the same ease with which we now graft and bud our grapes and peaches?

I fancy I foresee the day when the severed jugular vein or carotid artery shall have such a delicate adjustment of compression applied above and below the cleavage as to restrain an otherwise fatal hemorrhage and permit of the restoration of severed continuity by some more delicate and masterly stitch than the Czerny-Lembert suture.

In my fancy I catch fore-gleams of the day when some more glorious Jackson or Simpson shall stretch us on our dream couch with some new anæsthetic as free from harm and danger to all mankind and as happy in effect as now the cataleptic sleep is to a few.

Or, shall not some Charcot find the magic wand by which all men shall then be charmed to lethal slumbers for the surgeon's work without the use of drugs at all?

The hopeful prophet in our ranks can foresee the day when the hypnotized patient shall sit in the surgeon's chair and tell stories while his brain is being mended, as now he sits in the barber's chair to have his scalp tonsorialized, and thoracoplasty and craniosection shall come to be an art that needs no mending.

If we may justly judge the future by the past, we are to-day warranted in

> "Dreaming dreams no mortal ever dared to dream before."

We are warranted in exercising a high and long range prevision, warranted in foreseeing and foretelling events that shall come to pass in distant days.

We are warranted in multiplying achievements of this century by all of the achievements of past ages, and foretelling the day when Velpeau shall no longer be called the "King of Surgeons," and Senn and Helmuth shall seem as tyros to the men on yonder height.

Darwin proclaimed *The Descent of Man*, Drummond proclaimed *The Ascent of Man* and man himself proclaims his own rapid and unending progression,

> Over the bounds of that shadowy verge
> Where the known and the unknown meet and merge.

Just on the eve of the twentieth century there is breaking a glorious morning containing a new ray of light by which,

> We see in outlines, flesh-bereft
> The scaffolding that Nature left
> Within the temple on the day
> She housed the soul of man in clay.
> And with a new and wondrous art
> We trace the chambers of the heart,
> And watch with eager eyes the flow
> Of purple currents to and fro,
> And see the flood-gates rise and fall
> Beyond the temple's inner wall.
> We trace the new and rising zone
> As Nature knits the broken bone.
> While through the labarynthine mesh
> Of bone and sinew, nerve and flesh,
> We follow, like the hunter's pack,
> Swift-footed on the bullet's track.
> And with these mortal eyes profane
> The inner sanctum of the brain,
> And walk unhindered through the whole
> Majestic palace of the soul.

